# The Possibilities of Multiparametric Magnetic Resonance Imaging to Reflect Functional and Structural Graft Changes 1 Year After Kidney Transplantation

**DOI:** 10.3390/medicina61071268

**Published:** 2025-07-13

**Authors:** Andrejus Bura, Gintare Stonciute-Balniene, Laura Velickiene, Inga Arune Bumblyte, Ruta Vaiciuniene, Antanas Jankauskas

**Affiliations:** 1 Nephrology Department, Lithuanian University of Health Sciences, 44307 Kaunas, Lithuania; andrejus.bura@lsmu.lt (A.B.); ingaarune.bumblyte@lsmu.lt (I.A.B.); 2 Radiology Department, Lithuanian University of Health Sciences, 44307 Kaunas, Lithuania; gintare.stonciute@lsmu.lt (G.S.-B.); laura.velickiene@lsmu.lt (L.V.); antanas.jankauskas@lsmu.lt (A.J.)

**Keywords:** chronic graft nephropathy, interstitial fibrosis and tubular atrophy, renal transplantation, magnetic resonance imaging, non-invasive imaging biomarkers

## Abstract

*Background and Objectives*: Non-invasive imaging biomarkers for the early detection of chronic kidney allograft injury are needed to improve long-term transplant outcomes. T1 mapping by magnetic resonance imaging (MRI) has emerged as a promising method to assess renal structure and function. This study aimed to determine the potential of MRI as a diagnostic tool for evaluating graft function and structural changes in kidney grafts 1 year after transplantation. *Materials and Methods*: Thirty-four kidney transplant recipients were prospectively recruited, with 27 completing the follow-up at one year. Renal MRI at 3T was performed to acquire T1, T2, and apparent diffusion coefficient (ADC) maps. Clinical parameters, including estimated glomerular filtration rate (eGFR), albumin-to-creatinine ratio (ACR), protein-to-creatinine ratio (PCR), and histological IF/TA scores, were collected. MRI parameters were compared across the groups stratified by clinical and histological markers. Diagnostic accuracy was assessed using receiver operating characteristic (ROC) analysis. *Results*: At 1 year, T1 corticomedullary differentiation (CMD) values were significantly higher in patients with elevated ACR (≥3 mg/mmol), PCR (≥15 mg/mmol), and mild to moderate or severe IF/TA, reflecting a reduction in the corticomedullary gradient. T1 CMD demonstrated moderate-to-good diagnostic performance in detecting ACR (AUC 0.791), PCR (AUC 0.730), and IF/TA (AUC 0.839). No significant differences were observed in T2 or ADC values across these groups. T1 CMD also showed a significant positive correlation with ACR but not with eGFR, suggesting a closer association with structural rather than functional deterioration. *Conclusions*: T1 mapping, particularly T1 CMD, shows promise as a non-invasive imaging biomarker for detecting chronic allograft injury and monitoring renal function 1 year after kidney transplantation.

## 1. Introduction

Kidney transplantation (kTx) constitutes the most efficacious therapeutic intervention for individuals with end-stage chronic kidney disease (CKD) [1]. A primary objective for physicians is to identify high-risk patients with compromised allograft function early, enabling timely intervention and slowing disease progression.

Conventional measures such as estimated glomerular filtration rate (eGFR), albumin-to-creatinine ratio (ACR), and protein-to-creatinine ratio (PCR) have been criticized for capturing the outcomes of disease progression rather than identifying early pathological changes. However, PRC and ARC are well-recognized predictors of renal function decline in both transplant and non-transplant patients [2,3]. Previous studies have demonstrated that the presence of pathological albuminuria at various time points after kTx increases the risk of graft loss [2,4,5,6]. Despite these findings, there remains an unmet clinical need for novel diagnostic methods to detect chronic alterations in transplanted kidneys earlier.

Advances in proteomic and metabolomic profiling, RNA biomarker analysis, and detection of donor-derived cell-free DNA have shown encouraging potential for improving kidney graft monitoring. Biomarkers like urinary CXCL10 and donor-derived DNA can offer earlier and more specific insights into graft rejection and function than traditional markers such as serum creatinine [7,8]. Despite their potential, these biomarkers are still under investigation and have not yet been fully integrated into routine clinical practice.

To address this diagnostic gap, some centers perform protocol kidney biopsies [8]. The histological evaluation based on the Banff criteria for renal allograft pathology [9] provides important information on subclinical rejection, toxicity related to calcineurin inhibitors, and chronic graft damage, such as interstitial fibrosis and tubular atrophy (IF/TA) [10]. These findings aid in predicting graft survival and guiding clinical decisions to optimize long-term outcomes [11,12]. However, kidney biopsy remains an invasive procedure that is time-consuming and associated with potential complications: 3.18% (95% CI, 2.31–4.19%) for any major event, 0.31% (95% CI, 0.15–0.52%) for transfusion-requiring bleeding, and 0.89% (95% CI, 0.61–1.22%) for significant hematuria [13]. In addition, biopsy results can be affected by sampling error [10].

In response to these challenges, multiparametric magnetic resonance imaging (MRI) has gained attention as a promising non-invasive method for evaluating the structure and function of kidney allografts [14]. MRI provides valuable complementary information when integrated with clinical findings. Advanced MRI techniques, including T1 and T2 mapping, facilitate the evaluation of the renal molecular environment [14,15], while diffusion-weighted imaging (DWI) assesses tissue microstructure by measuring water molecule motion to generate the apparent diffusion coefficient (ADC) [15,16]. Notably, these imaging modalities eliminate the need for contrast agents, enabling safe, repeated assessments without complications.

T1 mapping has shown promise in identifying allograft rejection and acute tubular necrosis [17,18,19]. In our previous study, we demonstrated that cortical-medullary differentiation (CMD), assessed by T1 mapping at 10 to 15 days post-kTx, could predict the development of chronic allograft nephropathy (histologically defined as IF/TA) within three months [20]. Similar to other researchers, we also observed correlations between T1 mapping and eGFR [21,22,23,24,25]. Likewise, T2 mapping has shown prolonged relaxation times in transplanted kidneys, consistent with findings from other studies [22,26].

Early and intermediate post-transplant ADC values correlate with kidney function [20,22,24,27,28,29,30,31], and MRI sequences have shown associations with interstitial fibrosis in both transplanted and native kidneys [22,23,26,32,33]. The findings underscore the potential of MRI to non-invasively monitor graft function and predict future outcomes.

Study Aim: to determine the potential of MRI as a diagnostic tool for evaluating graft function and structural changes in a kidney graft 1 year after transplantation.

## 2. Materials and Methods

### 2.1. Study Design

The prospective observational cohort study enrolled recipients of deceased-donor kidney transplants at the Lithuanian University of Health Sciences Hospital Kauno Klinikos from May 2022 to December 2023. The City Region Biomedical Ethics Committee granted ethical approval (reference number BE-2-12, dated 22 February 2022), and written informed consent was obtained from all participants. We conducted this study to evaluate the structural MRI changes in kidney transplants concerning 1 year IF/TA, eGFR, ARC, and PRC. During the follow-up period, MRI scans were performed 1 year after kTx. The MRI examinations included T1 mapping, T2 mapping, and DWI. One year after the transplant, one day before the kidney biopsy, an MRI scan was performed. The biopsy specimens were assessed by a pathologist in accordance with the Banff 2022 classification criteria. At the same time, data about eGFR, ARC, and PRC were collected.

### 2.2. Study Population

During the study period, 40 deceased-donor kidney transplantations (kTx) were performed. Patients were excluded from analysis if they experienced primary non-function (PNF; n = 1), had claustrophobia preventing MRI (n = 5), or withdrew consent for social reasons (n = 7). PNF was defined as permanent graft failure immediately following transplantation. The final cohort comprised 27 patients who completed the 1-year follow-up.

Standard immunosuppressive regimens included induction therapy with Basiliximab or anti-thymocyte globulin for those at high immunological risk, followed by maintenance triple therapy with methylprednisolone, mycophenolate mofetil, and calcineurin inhibitors. Kidney allografts from deceased donors were preserved utilizing either cold storage immersion techniques or the LifePort Kidney Transporter device.

Comprehensive data were collected on recipient demographics and clinical variables, donor characteristics, and graft function at 12 months post-transplantation (Table 1). Extensive data on the recipients’ clinical profiles and the renoprotective medications administered over the one-year observation period are provided in the Appendix A (Table A1).

### 2.3. Evaluation of Graft Function

Patients were categorized based on eGFR at one-year after-kTx follow-up:

- eGFR ≥ 60 mL/min/1.73 m^2^ (n = 11) vs. <60 mL/min/1.73 m^2^ (n = 16).

According to ACR and PCR levels 1 year after-kTx, patients were categorized into the groups:

- ACR ≥ 3 mg/mmol (n = 13) vs. ACR <3 mg/mmol (n = 14);
- PCR ≥ 15 mg/mmol (n = 15) vs. PCR <15 mg/mmol (n = 12).

### 2.4. Evaluation of Renal Allograft Biopsies

Key structural changes were assessed by summing the Banff scores for interstitial fibrosis (ci) and tubular atrophy (ct), resulting in the IF/TA score (Table A2, Appendix A).

At 1 year after transplantation, the groups were classified according to IF/TA:

- No or minimal IF/TA (n = 7).
- Mild-to-moderate or severe IF/TA (n = 8).

### 2.5. MRI Imaging, Protocol and Analysis

MRI scans were acquired on a 3T MAGNETOM Skyra system (Siemens, München, Germany) with a 32-channel coil, following standard guidelines [15,16]. Localization: HASTE sequences identified the transplanted kidney. T1/T2 Mapping: Three coronal slices (8 mm) were acquired using MOLLI for T1 and SSFP for T2 mapping with motion correction. DWI: Axial scans used a free-breathing echo-planar technique with b-values of 0, 100, 200, and 800 s/mm^2^. MRI data were analyzed using PMRI software (Philadelphia, PA, USA, V1.0). ADC maps were generated via a mono-exponential model, and T1/T2 maps were assessed. Two independent observers (radiologist and nephrologist) delineated cortical and medullary ROIs (Appendix B). Measurements were averaged, and corticomedullary ratios (CMD) were calculated. Detailed methodological information can be found in our prior article [20].

### 2.6. Statistical Analyses

Statistical analyses were performed using IBM SPSS Statistics version 30.0.0.0 (IBM Corp., Armonk, NY, USA). Continuous variables were summarized as means with standard deviations (SDs) or medians with interquartile ranges (IQRs), depending on data distribution. Categorical variables were presented as counts and percentages.

Comparisons of continuous variables, including T1 and T2 mapping values, ADC, and clinical parameters, were conducted using the Mann–Whitney U test for data not following a normal distribution. Relationships between continuous variables were evaluated using Spearman’s rank correlation coefficient.

Receiver operating characteristic (ROC) curve analyses were utilized to determine the predictive performance of T1 CMD at one year post-kidney transplantation for detecting elevated ACR (≥3mg/mmol), PCR (≥15mg/mmol), and mild-to-moderate or severe IF/TA.

Interobserver agreement between the radiologist and nephrologist was assessed via the intraclass correlation coefficient (ICC), with values above 0.75 considered indicative of good reliability (see Appendix B). All statistical tests were two-tailed, and significance was set at *p* < 0.05.

## 3. Results

### 3.1. Patient Characteristics and Clinical Findings in the Follow-Up Post-Transplant Period

During the 1-year follow-up period, 27 patients remained in this study. Demographic and clinical data are presented in Table 1.

### 3.2. MRI Values According to the eGFR Groups 1 Year After Transplantation

**Figure 1:**
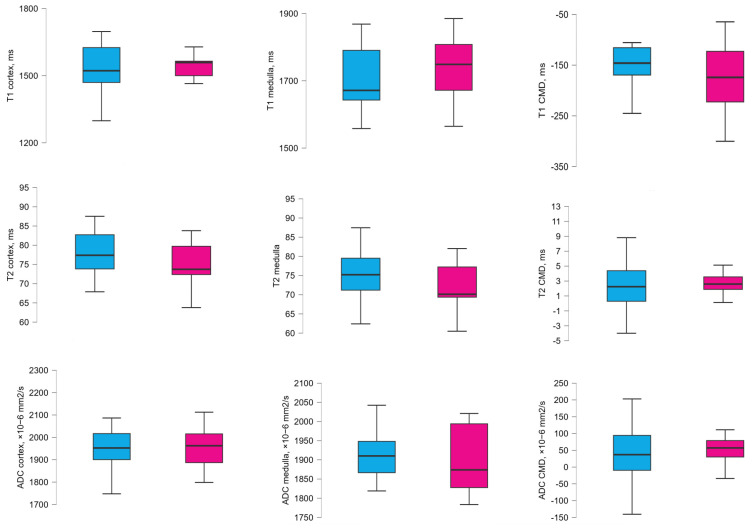
Mean and distribution of T1, T2, and ADC cortical and medullary values in 2 eGFR groups at 1 year after transplantation. The blue bar −eGFR ≥ 60 mL/min/1.73 m^2^, and the red bar −<60 mL/min/1.73 m^2^.

MRI-derived T1, T2 mapping values and apparent diffusion coefficient (ADC) measurements at 1 year after transplantation did not show statistically significant differences between the eGFR groups. Detailed analyses are presented in Appendix C (Table A3 and Figure 1).

Spearman’s correlation analysis did not reveal any significant correlation between eGFR and MRI parameters across all sequences at 1 year after kidney transplantation.

### 3.3. MRI Values According to the ACR Groups 1 Year After Transplantation

**Figure 2:**
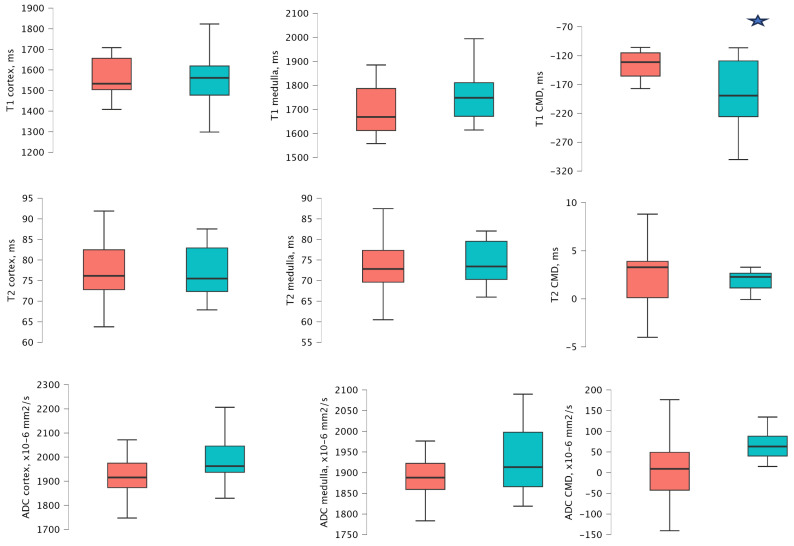
Mean and distribution of T1, T2, and ADC cortical and medullary values in 2 ACR groups 1 year after transplantation. The red bar − ACR ≥ 3 mg/mmol, and the green bar − ACR < 3 mg/mmol. The star icon indicates significant differences.

At 1 year after transplantation, patients with an ACR ≥ 3 mg/mmol demonstrated significantly higher T1 CMD values compared to those with ACR < 3 mg/mmol values (−130.55 ms [−146.69 to −106.96 ms] vs. −189.19 ms [−255.65 to −129.01 ms], *p* = 0.009). Other MRI sequences did not reveal significant differences. Detailed analyses are provided in Appendix D, Table A4 and Figure 2.

**Figure 3:**
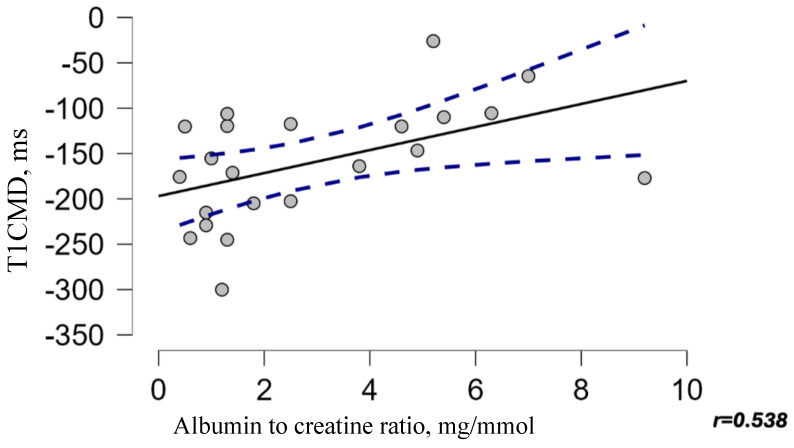
Correlation between the albumin to creatinine ratio and T1 CMD at 1 year after kTx. The blue dashed lines represent the 95% confidence interval.

A moderate positive correlation was identified using Spearman’s rank correlation between T1 CMD values at 1 year after-kTx and ACR level 1 year after-kidney transplantation (ρ = 0.538, *p* = 0.010, Figure 3). There was no statistically significant correlation between albuminuria and additional MRI sequences.

### 3.4. MRI Values According to the PCR Groups 1 Year After Transplantation

**Figure 4:**
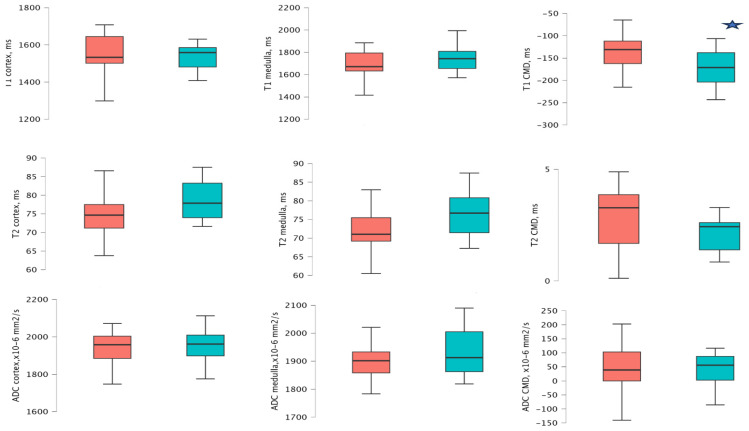
Mean and distribution of T1, T2, and ADC cortical and medullary values at follow-up in 2 PCR groups 1 year after transplantation. The red bar − PCR ≥ 15 mg/mmol, and the green bar − PCR < 15 mg/mmol. The star icon indicates significant differences.

Patients with a PCR ≥ 15 mg/mmol at 1 year demonstrated significantly higher T1 CMD values compared to those with PCR < 15 mg/mmol (−130.55 ms [−157.05 to −108.44 ms] vs. −173.49 ms [−210.95 to −146.60 ms], *p* = 0.047). Other MRI sequences did not reveal significant differences. Detailed analyses are provided in Appendix E, Table A5, and Figure 4.

No significant correlations were found between PCR level and other MRI sequences during the follow-up period.

### 3.5. MRI Values According to the IF/TA Groups 1 Year After Transplantation

**Figure 5:**
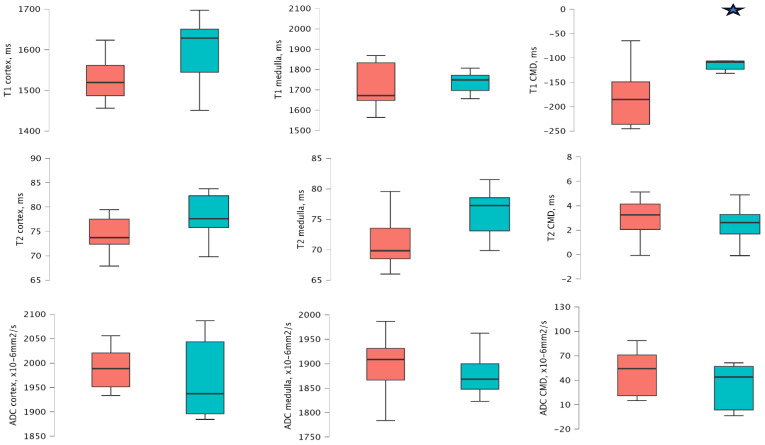
Mean and distribution of T1, T2, and ADC cortical and medullary values in IF/TA groups at 1 year after transplantation. The red bar − no or minimal IF/TA, and the green bar − mild-to-moderate or severe IF/TA. The star icon indicates significant differences.

Consistent with the ACR and PCR groups, at 1 year, patients with mild-to-moderate or severe IF/TA exhibited significantly higher T1 CMD compared to those with no or minimal IF/TA (−215.16 [−244.14 to −151.04 ms] vs. −108.44 ms [−122.96 to −106.10 ms], *p* = 0.029). The other MRI sequences did not demonstrate significant differences. Detailed analyses are provided in Appendix F, Table A6, and Figure 5.

### 3.6. Diagnostic Value of MRI T1 Map Parameters for Detecting ACR, PCR, and IF/TA 1 Year After kTx

The T1 CMD at 1 year after kTx demonstrated favorable diagnostic performance, with an AUC of 0.791 [0.618–0.965] for identifying ACR ≥ 3 mg/mmol, an AUC of 0.730 [0.533–0.923] for PCR ≥ 15 mg/mmol, and an AUC of 0.839 [0.601–1.077] for diagnosing mild-to-moderate or severe IF/TA.

**Figure 6:**
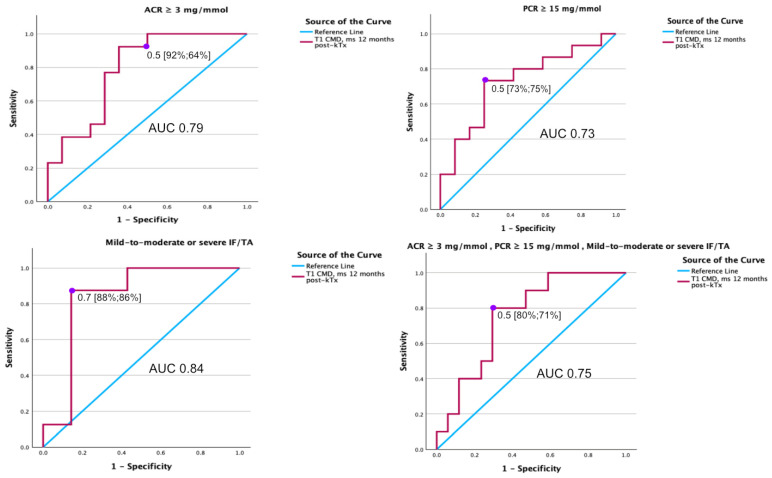
The diagnostic accuracy of T1 CMD measured one year after kidney transplantation is illustrated by the receiver operating characteristic curve (red line), with the purple dot indicating the optimal cutoff based on Youden’s index, for detecting abnormalities in the transplanted kidney at the same time point.

Overall, the T1 CMD at 1 year after kTx exhibited an AUC of 0.753 [0.570–0.936] for diagnosing all pathologies combined (Table 2 and Figure 6).

## 4. Discussion

This study demonstrates the potential of T1 mapping as a non-invasive and reliable tool for assessing chronic renal injury after kidney transplantation. The significant associations observed between T1 CMD values and markers of graft dysfunction, such as ACR, PCR, and IF/TA, highlight the potential of its diagnostic utility.

However, we observed that both T1 and ADC mapping lost their correlation with eGFR at 1 year after kidney transplantation. This finding is in line with previous studies suggesting that variations in follow-up duration, cohort size, or the presence of MRI artefacts during post-processing [34] may affect these correlations [35]. Aurelie Huber reported a weak correlation between T1 values and eGFR (cortical T1: R = −0.1, *p* = 0.197; medullary T1: R = 0.2, *p* = 0.017; ΔT1: R = −0.3, *p* < 0.001) [36]. Similarly, Buchanan et al. [37] described persistent structural abnormalities measured by T1 mapping in patients who had recovered from acute kidney injury, even when their biochemical kidney function appeared normal. These observations support the idea that raised T1 values represent a combination of histological features, including tubular atrophy and interstitial inflammation, rather than being specific to fibrosis alone [38,39], which may be reversible and influence their association with renal function. Therefore, for a more comprehensive assessment of renal function, there may be a need for complementing T1 mapping with additional MRI sequences, such as arterial spin labelling (ASL), diffusion-weighted imaging (DWI), blood oxygen level-dependent (BOLD) R2 mapping, or renal blood flow imaging [35,37,40].

Proteinuria, although a non-specific marker of glomerular filtration barrier dysfunction, remains clinically important. A previous study investigating multiparametric MRI in CKD patients reported a moderate positive correlation between T1 CMD values and PCR (r = 0.61) [41]. Mao et al. [42] observed an inverse correlation between diffusion metrics and proteinuria, but this finding did not replicate in our study. Nonetheless, our results align with earlier reports, showing increased T1 CMD values in transplant recipients with pathological albuminuria and proteinuria, further supporting the role of T1 mapping in detecting early structural kidney damage.

In vivo measurements of renal T2 in humans remain limited in the current literature. Consistent with previous studies [22,26], we observed a persistently prolonged T2 relaxation time that did not decrease even 1 year after kTx [20]. Similar to findings by Beck-Tölly, Andrea [43], our T2 map values did not correlate with histological findings.

Interstitial fibrosis and tubular atrophy, as the final common pathway of diverse immunological and non-immunological insults, are key indicators of chronic kidney damage [44,45]. As observed in our previous work [20], recipients with mild-to-moderate or severe IF/TA showed greater T1 CMD values and a trend toward increased cortical and medullary T1 values. This pattern was also reported by Chao-Gang Wei et al. [46], who demonstrated that the severity of fibrosis in CKD patients was associated with reduced T1 CMD. Similarly, studies by Beck-Tölly, Andrea, and Wei Mao [38,43] found that cortical T1 values correlate with tubulointerstitial injury scores, a finding consistent with our results on IF/TA. In the mild-to-moderate or severe IF/TA group, prolonged cortical T1 values likely contributed to the observed reductions in T1 CMD. Additionally, we noted a significant enhancement in T1 CMD values and an increasing trend in cortical and medullary T1 values, along with worsening CMD on ADC maps in fibrotic transplanted kidneys, echoing the findings of Aurelie Huber [36].

Our study demonstrates the diagnostic value of T1 CMD at 1 year after transplant. We found that T1 CMD alone exhibited moderate-to-good diagnostic performance in identifying ACR (AUC 0.791), PCR (AUC 0.730), and IF/TA (AUC 0.839). Among patients with elevated ACR and PCR, 12 out of 27 (44.44%) overlapped. Across the full cohort, one patient presented with isolated albuminuria, and three had isolated non-selective proteinuria. In the subgroup with mild-to-moderate or severe IF/TA, five of eight patients had ACR ≥ 3 mg/mmol, and five had PCR ≥ 15 mg/mmol, and all three measures (IF/TA mild to moderate or severe, PCR ≥ 15 mg/mmol and ACR ≥ 3 mg/mmol) overlap in three patients, indicating partial—but not complete—overlap between functional and structural markers. We acknowledge that this overlap may confound the interpretation of T1 CMD specificity, as functional and structural impairments often co-exist in clinical settings. While this reflects real-world complexity, it limits our ability to attribute T1 CMD changes to a single pathological domain. Due to the limited sample size, we did not perform formal subgroup interaction analyses. Nonetheless, the overall diagnostic performance of T1 CMD for detecting any pathological findings was acceptable (AUC 0.753), supporting its possibility to use of T1 CMD as a non-invasive imaging biomarker for detecting graft pathology at a single time point post-transplant.

Unlike the earlier study [38,47], which focused on the relationship between T1 values and longitudinal renal function decline, our analysis emphasizes the cross-sectional diagnostic potential of T1 CMD for detecting established pathological lesions. This distinction between predicting future decline and diagnosing current pathology may help explain the stronger diagnostic performance observed in our study.

Berchtold et al. [48] demonstrated that T1 CMD correlates more strongly with eGFR and histological IF/TA in CKD patients than absolute cortical or medullary T1 values alone. Similar findings were reported in transplant recipients by Ibtisam Aslam et al. [49], supporting the relevance of T1 CMD in the post-transplant setting. Moreover, while previous research has noted the lack of specificity of cortical T1 values to fibrosis, highlighting their elevation in inflammation and tubular atrophy [38,39]. Our findings align with these observations, suggesting that T1 CMD—by incorporating both cortical and medullary tissue characteristics—may serve as a more robust and integrative biomarker of renal pathology and its progression than absolute T1 values alone.

This study is limited by a relatively small sample size of 27 patients, with even smaller subgroups, which may reduce the generalizability of the results. Unlike the findings reported previously [43], our analysis did not reveal a significant relationship between interstitial fibrosis and either T1 or ADC values. Furthermore, we were unable to perform renal BOLD and ASL imaging, which could have provided additional functional information. Our study’s monocentric nature poses limitations on the wider relevance of the findings. Nevertheless, the study cohort showed no signs of subclinical or acute rejection at the 1-year protocol biopsy, which allowed us to evaluate the relationship between MRI parameters and IF/TA progression without confounding factors. This homogeneity, while beneficial for internal validity, may limit the possibilities for wider use of MRI in diagnosing early post-transplant complications, which can also contribute to IF/TA through inflammatory mechanisms. These possibilities need testing in further studies.

## 5. Conclusions

T1 mapping, particularly T1 corticomedullary differentiation, shows promise as a non-invasive imaging biomarker for detecting chronic allograft injury and monitoring renal function 1 year after kidney transplantation.

## Figures and Tables

**Table 1:** Demographic and clinical data one year after kidney transplantation.

Recipients	One Year After Transplant
	n = 27
Gender male (%)	18 (66.70)
Age (years)	43.00 (34.00–55.00)
Duration of kidney replacement therapy (months)	16.00 (6.00–43.00)
HLA mismatch	3 (3–4)
Kidney disease (%)	Chronic glomerulonephritis: 4 case (14.8%) Diabetic nephropathy: 1 case (3.7%) Autosomal dominant polycystic kidney disease: 4 cases (14.8%) Hypertensive nephropathy: 2 cases (7.4%) Other: 16 cases (59.3%)
Immunosuppression regimen	Methylprednisolone: 100% Mycophenolate mofetil: 100% Tacrolimus: 100% Induction therapy: Anti-thymocyte globulin: 3 cases (11.1%) INN-basiliximab: 24 cases (88.9%)
Creatinine before kTx (μmol/L)	738.00 (507.00–952.00)
eGFR at discharge day (mL/min/1.73 m^2^)	61.00 (46.00–79.00)
eGFR at 3 months post kTx (mL/min/1.73 m^2^)	49.10 (45.40–68.10)
eGFR at 12 months post kTx (mL/min/1.73 m^2^)	57.90 (49.70–68.40)
Albumin to creatinine ratio at 12 months post kTx (mg/mmol)	2.50 (1.20–6.30)
Protein to creatinine ratio at 12 months post kTx (mg/mmol)	15.70 (9.20–29.20)
Donors	
Age (years)	54.00 (46.00–61.00)
Expanded criteria donor ^1^ (%)	10 (37.00)
Cold ischemia time of transplanted kidney (min)	720 (656.00–900.00)

^1^ Expanded criteria donor—60 years and older, or more than 50 years old with two criteria: arterial hypertension, serum creatinine > 130 μmol/L, cerebrovascular cause of death. Data are given as a number (%), mean (SD) or median (IQR).

**Table 2:** Diagnostic efficiency of MRI parameters for detecting complication 1 year after kTx follow-up.

MRI Parameters					
ACR ≥ 3 mg/mmol					
	AUC	Cl I95%)	Sensitivity (%)	Specificity (%)	*p*-value
T1 cortex	0.52	0.287–0.746			0.888
T1 medulla	0.36	0.148–0.577			0.209
T1 CMD	0.79	0.618–0.965	92	64	0.001
PCR ≥ 15 mg/mmol					
T1 cortex	0.50	0.269–0.719			0.961
T1 medulla	0.40	0.178–0.611			0.339
T1 CMD	0.73	0.533–0.923	73	75	0.022
Mild-to-moderate or severe IF/TA					
T1 cortex	0.66	0.360–0.961			0.295
T1 medulla	0.39	0.080–0.705			0.502
T1 CMD	0.84	0.601–1.077	88	86	0.005
ACR ≥ 3 mg/mmol PCR ≥ 15 mg/mmol mild-to-moderate or severe IF/TA					
T1 cortex	0.62	0.379–9.856			0.333
T1 medulla	0.48	0.247–0.718			0.883
T1 CMD	0.75	0.570–0.936	80	71	0.007

## Data Availability

The data presented in this study are available upon request from the corresponding author. They are not publicly available due to limited ethical approval.

## Abbreviations

The following abbreviations are used in this manuscript:

kTx	Kidney transplantation
CKD	Chronic kidney disease
IF/TA	Interstitial fibrosis and tubular atrophy
MRI	Magnetic resonance imaging
ADC	The apparent diffusion coefficient
eGFR	Estimated glomerular filtration rate
ARC	Albumin to creatinine ratio
PRC	Protein to creatinine ratio
PNF	Primary non-function
MRR	Magnetic resonance relaxometry
MOLLI	A modified Look–Locker inversion recovery
SSFP	A single-shot balanced steady-state free precession
DWI	Diffusion-weighted imaging
CMD	Corticomedullary differentiation
SD	Standard deviation
IQR	Interquartile range
CI	95% confidence intervals
ROC	Receiver operating characteristic (ROC)
ICC	The intraclass correlation coefficient

## Appendix A

**Table A1:** Clinical characteristics and renoprotective medications administered to kidney transplant recipients at 1-year follow-up.

Characteristics	n (Pathology/Total)
History of acute and chronic recurrent pyelonephritis	9/27
Cytomegalovirus infection	1/27
Polyomavirus viremia	3/27
Hospital administration (another reasons)	5/27
Medication	
Renin–angiotensin system inhibitor	3/27
Angiotensin receptor blocker	5/27
Sodium-glucose cotransporter 2 inhibitor	1/27

### Appendix A.1. KTx Biopsy Evaluation

During the follow-up period, 15 protocol biopsies were conducted. One patient was excluded due to anticoagulant therapy, two patients experienced recurrent urinary tract infections, and nine patients declined to undergo biopsy. All biopsy specimens were evaluated at the National Center of Pathology.

**Table A2:** Biopsy data.

	1 Year After kTx Biopsy	
	Mean (SD)	N (Total/Pathology)
Glomerular count	30.40 (14.37)	15/15
Glomerular sclerosis, score	1.00 (1.31)	15/7
Glomerulitis, score	0.67 (0.82)	15/7
Inflammatory infiltrates	0.00 (0.00)	15/0
Peritubulatcapillaritis, score	0.20 (0.41)	15/3
Interstitial fibrosis, score	0.80 (0.86)	15/9
Tubular atrophy, score	0.87 (0.74)	15/11
Tubulitis, score	0.13 (0.35)	2/15
Arteriolar hyalinosis, score	0.66 (0.62)	15/9
IF/TA* score	1.67 (1.54)	15/11

Due to the poor representativeness of the median (IQR), we report only the mean (SD) to provide a more comprehensive understanding of the distribution. IF/TA*—interstitial fibrosis and tubular atrophy.

## Appendix B

### Appendix B.1. Imagine Analyses

- Image analyses were independently conducted by a radiologist and a nephrologist. A comprehensive description of the methodology is provided in our previous publication [20]. The intraclass correlation coefficients (ICCs) assessing agreement between the two specialists for kidney transplants (kTx) at 1-year post-transplantation were as follows: T1 map, cortical: 0.971 (95% CI 0.920–0.988); medulla: 0.955 (95% CI 0.877–0.981) (*p* < 0.001).
- T2 map, cortical: 0.986 (95% CI 0.970–0.994); medulla: 0.979 (95% CI 0.943–0.991) (*p* < 0.001).
- ADC, cortical: 0.956 (95% CI 0.898–0.980); medulla: 0.866 (95% CI 0.709–0.938) (*p* < 0.001).

## Appendix C

**Table A3:** Comparison of magnetic resonance imaging parameters between groups stratified by 1-year post-kTx graft function.

	eGFR ≥ 60 mL/min/1.73 m^2^	eGFR < 60 mL/min/1.73 m^2^	*p*-Value
Recipients	n = 11	n = 16	
T_1_ map of cortex (ms)	1558.53 (1497.45–1596.67)	1522.00 (1469.68–1625.41)	0.716
T_1_ map of medulla (ms)	1748.76 (1643.18–1833.32)	1668.72 (1628.56–1787.13)	0.451
T_1_ map of CMD ^1^ (ms)	−171.19 (−215.87(–)−119.94)	−146.04 (−169.50(–)−115.59)	0.544
T_2_ map of cortex (ms)	73.73 (72.37–79.72)	77.39 (73.84–82.73)	0.451
T_2_ map of the medulla (ms)	70.13 (69.36–77.24)	75.22 (71.19–79.52)	0.272
T_2_ map of CMD ^1^ (ms)	2.60 (1.88–3.56)	2.24 (0.286–4.39)	0.693
ADC value of cortex (×10^−6^ mm^2^/s)	1962.50 (1887.00–2015.50)	1952.50 (1900.50–2016.88)	0.824
ADC value of medulla (×10^−6^ mm^2^/s)	1874.00 (1827.75–1994.00)	1910.25 (1866.63–1948.25)	0.512
ADC CMD ^1^ (×10^−6^ mm^2^/s)	57.00 (30.00–79.00)	37.00 (−9.63–94.38)	0.521

^1^ Corticomedullary differentiation (CMD) is based on the following formula: cortical value–medulla value. Data are given as median (IQR).

## Appendix D

**Table A4:** Comparison of MRI measurements across groups defined by albumin-to-creatinine ratio one year after kTx.

	ACR < 3 mg/mmol	ACR ≥ 3 mg/mmol	*p*-Value
Recipients	n = 14	n = 13	
T_1_ map of cortex (ms)	1561.44 (1477.67–1619.28)	1524.68 (1500.01–1651.92)	0.905
T_1_ map of medulla (ms)	1743.02 (1665.90–1810.56)	1666.02 (1572.19–1783.74)	0.239
T_1_ map of CMD ^1^ (ms)	−189.19 (−255.65(–)−129.01)	−130.55 (−146.69(–)−106.96)	0.009
T_2_ map of cortex (ms)	75.50 (72.35–82.93)	76.16 (72.81–82.48)	0.981
T_2_ map of the medulla (ms)	73.43 (70.28–79.54)	74.30 (69.83–77.59)	0.943
T_2_ map of CMD ^1^ (ms)	2.43 (1.22–3.13)	3.28 (0.12–3.90)	0.961
ADC value of cortex (×10^−6^ mm^2^/s)	1962.50 (1937.70–2045.75)	1915.50 (1873.50–1975.00)	0.094
ADC value of medulla (×10^−6^ mm^2^/s)	1913.50 (1866.13–1997.75)	1888.00 (1859.50–1922.50)	0.239
ADC CMD ^1^ (×10^−6^ mm^2^/s)	63.25 (40.25–88.13)	15.00 (−28.00–61.50)	0.225

^1^ Corticomedullary differentiation (CMD) is based on the following formula: cortical value–medulla value. Data are given as median (IQR).

## Appendix E

**Table A5:** Comparison of MRI measurements across groups defined by protein-to-creatinine ratio one year after kTx.

	PCR < 15 mg/mmol	PCR ≥ 15 mg/mmol	*p*-Value
Recipients	n = 12	n = 15	
T_1_ map of cortex (ms)	1561.44 (1484.73–1612.03)	1524.68 (1478.14–1637.73)	0.981
T_1_ map of medulla (ms)	1743.02 (1655.43–1808.87)	1671.43 (1595.15–1790.52)	0.373
T_1_ map of CMD ^1^ (ms)	−173.49 (−210.95(–)−146.60)	−130.55 (−157.05(–)−108.44)	0.047
T_2_ map of cortex (ms)	77.87 (73.97–83.25)	75.56 (71.19–80.05)	0.347
T_2_ map of the medulla (ms)	76.69 (71.46–80.82)	71.34 (69.41–76.50)	0.217
T_2_ map of CMD ^1^ (ms)	2.43 (1.06–2.82)	3.28 (0.94–4.06)	0.435
ADC value of cortex (×10^−6^ mm^2^/s)	1961.75 (1901.88–2063.63)	1957.50 (1884.25–2003.50)	0.510
ADC value of medulla (×10^−6^ mm^2^/s)	1912.50 (1862.88–2005.25)	1901.50 (1858.50–1933.00)	0.323
ADC CMD ^1^ (×10^−6^ mm^2^/s)	55.75 (2.75–87.38)	39.00 (0.00–103.00)	0.961

^1^ Corticomedullary differentiation (CMD) is based on the following formula: cortical value–medulla value. Data are given as median (IQR).

## Appendix F

**Table A6:** MRI data comparison according to IF/TA grade at 1 year post-kidney transplantation.

	No or Minimal Fibrosis/Tubular Atrophy	Mild-to-Moderate or Severe Fibrosis/Tubular Atrophy	*p*-Value
Recipients	n = 7	n = 8	
T_1_ map of cortex (ms)	1519.33 (1487.08–1561.66)	1596.44 (1506.25–1640.87)	0.336
T_1_ map of medulla (ms)	1671.43 (647.78–1833.32)	1743.02 (1631.40–1769.20)	0.536
T_1_ map of CMD ^1^ (ms)	−215.16 (−244.14(–)−151.04)	−108.44 (−122.96(–)−106.10)	0.029
T_2_ map of cortex (ms)	73.73 (72.37–77.52)	77.39 (73.32–82.25)	0.613
T_2_ map of the medulla (ms)	69.83 (68.51–73.55)	75.78 (71.40–78.07)	0.232
T_2_ map of CMD ^1^ (ms)	3.90 (2.24–4.68)	2.63 (1.69–3.929)	0.336
ADC value of cortex (×10^−6^ mm^2^/s)	1962.50 (1940.50–2018.75)	1902.50 (1870.75–987.13)	0.463
ADC value of medulla (×10^−6^ mm^2^/s)	1908.50 (1866.75–1931.00)	1878.25 (1855.75–1924.63)	0.867
ADC CMD ^1^ (×10^−6^ mm^2^/s)	69.50 (27.00–80.00)	23.75 (−11.13–58.13)	0.183

^1^ Corticomedullary differentiation (CMD) is based on the following formula: cortical value–medulla value. Data are given as median (IQR).
